# Tumor Patients´ Perceived Changes of Specific Attitudes, Perceptions, and Behaviors Due to the COVID-19 Pandemic and Its Relation to Reduced Wellbeing

**DOI:** 10.3389/fpsyt.2020.574314

**Published:** 2020-10-09

**Authors:** Arndt Büssing, Jutta Hübner, Stefanie Walter, Wolfgang Gießler, Jens Büntzel

**Affiliations:** ^1^Professorship Quality of Life, Spirituality and Coping, Witten/Herdecke University, Herdecke, Germany; ^2^Working Group “Prevention and Integrative Oncology” (PRIO) in the German Cancer Society, Berlin, Germany; ^3^Professorship Integrative Oncology, Medical Clinic II, University Clinic Jena, Jena, Germany; ^4^Bundesverband der Kehlkopfoperierten e. V., Bonn, Germany; ^5^Department Hematology/Oncology and Palliative Medicine, Clinic Wetzlar, Wetzlar, Germany; ^6^Department of Otolaryngology, Palliative Care Unit, Südharz Clinic Nordhausen, Nordhausen, Germany

**Keywords:** tumor patients, elderly, corona pandemic, wellbeing, change of attitudes, spirituality, meaning in life, COVID-19

## Abstract

**Background:**

During the COVID-19 pandemic, the Working Group “Prevention and Integrative Oncology” (PRIO) in the German Cancer Society has initiated flash interviews and surveys. One of these stated increasing rates of fears and mental stress of tumor patients. Now we aimed to analyze whether tumor patients did perceive changes in their attitudes and behaviors related to their relationships, awareness of nature and quietness, interest in spiritual issues, or feelings of worries and isolation. A further point of interest was how these perceived changes could be predicted, either by meaning in life, spirituality as a resource to cope, perceived fears and worries, or particularly by their wellbeing.

**Materials and Methods:**

Online survey with standardized questionnaires (i.e., WHO-Five Well-being Index (WHO5), Meaning in Life Questionnaire (MLQ), Spiritual and Religious Attitudes to cope with illness (SpREUK-15), Gratitude/Awe scale (GrAw-7)) among 292 tumor patients (72% men; mean age 66.7 ± 10.8 years; 25% < 60 years, 33% 60-70 years, 41% > 70 years) from Germany between May 6 to June 10, 2020.

**Results:**

Patients´ wellbeing (WHO5) scores were in the lower range (14.7 ± 6.0); 35% scored < 13, indicating depressive states. Wellbeing was significantly higher in older persons and lower in younger ones (F=11.1, p<.0001). Most were irritated by different statements about the danger and the course of the corona infection in the public media (60%), and 57% were worrying to be infected and to have a complicated course of disease. Because of the restrictions, patients noticed changes in their attitudes and behaviors (measured with the 12-item *Perceptions of Change Scale*): 1) *Perception of nature and silence* (Cronbach´s alpha = .82), 2) *Worrying reflections and loneliness* (Cronbach´s alpha = .80), 3) *Interest in spirituality* (Cronbach´s alpha = .91), 4) *Intense relationships* (Cronbach´s alpha = .64). These perceptions of change were similar in women and men, age groups and also with respect to tumor stages. Regression analyses revealed that the factor *Perception of nature and silence* was predicted best by patients´ ability to value and experience the ‘wonder’ of the present moment (in terms of wondering awe and gratitude) and by patients´ search for meaning in life. The factor *Worrying reflections and loneliness* was predicted best by their search for meaning in life and by feelings of being under pressure because of the Corona pandemic. *Interest in spirituality* was predicted best by search for an access to a spiritual source and by frequency of praying. *Intense relationships* were explained with weak predictive power by patients´ ability to reflect life concerns. Patients´ wellbeing during the Corona pandemic was predicted (R^2^ =.57) by a mix of disease and pandemic related stressor, and by available resources (meaning in life and religious trust).

**Conclusion:**

In this study among tumor patients from a secular society the topics meaning in life, having (religious) trust, stable relationships, mindful encounter with nature, and times of reflection were found to be of importance. To overcome tumor patients´ feelings of isolation, depressive states, and insecurity about future perspectives, further support is needed, particularly in their socio-spatial surrounding. These are the domains of psychotherapy and spiritual care. The planned integration of structured access to spiritual care seems to be important, not only for the field of cancer care. As the findings refer to patients´ self-perceptions, longitudinal studies are required to substantiate these perceived changes.

## Introduction

The COVID-19 pandemic resulted in a societal lockdown in Germany. The public health system has focused on preventive strategies and treatment possibilities for infected patients at ICU and normal hospitals. Most persons in Germany followed the individual and social restrictions and stayed at home. As a consequence, several felt isolated from their friends and relatives, missed the collaborative networks at their job, and had to deal with so much (boring) ‘extra time’. Some experienced fears to get in contact with potentially infected persons, avoided direct contact with others, and were allowed to go to the grocery store and pharmacies only. Several patients with chronic diseases and also those with acute illness symptoms avoided going to hospitals or meeting their medical doctors, because they feared potential infection routes.

The Working Group “Prevention and Integrative Oncology” (PRIO) in the German Cancer Society has initiated a series of flash interviews among the stakeholders during the crisis in order to reflect the moment and to develop strategies to be better prepared for next critical situations. These flash interviews have documented increasing rates of fear and mental stress of tumor patients and their physicians during the crisis in April 2020 (1). Main problems were common anxieties regarding delays, therapy breaks or finishing these treatments. A majority of patients reported diffuse fears of the future. Half of the oncologists and nurses were awaiting their own physical and/or mental burdens as a consequence of actual pandemic management. Similar data were reported by Italian colleagues, especially for patients suffering from both cancer and infection (2).

Apart from fears and worries, several persons anecdotally reported that they used the ‘extra time’ of the lockdown to spend more time outdoors, to perceive nature more intensely, to spend more time with their partner and their children, read more books, etc. - and generally to have more time for themselves. This ‘extra time’ could be used as a chance to reflect on those matters which may give meaning in life, to reflect what is essential in life, maybe also as a hint to change important aspects of life, to be more aware of nature and people around, and to deal more consciously (‘mindfully’) with them. Further, some may have experienced that these restricted times allowed them to focus more on their own interests instead on work related duties, and thus some may have enjoyed the ‘silence’, while others feared this ‘silence’ because they became aware of their loneliness from which they could be distracted more easily through various duties. These perceived changes of attitudes and behaviors have two directions, internal and external directed changes.

The aim of the study was to analyze whether patients with malignant tumors during the COVID-19 pandemic perceived changes of their attitudes and behaviors related to their relationships, awareness of nature and quietness, interest in spiritual issues, or feelings of worries and isolation. Tumor patients´ higher ‘vulnerability’ (i.e., worries about the course of their disease, fear of relapse, avoidance of routine visits because of their insecurity about potentially infection routes during the COVID-19 pandemic) may have resulted in more differentiated views which can be seen as reappraisal coping strategies (3) as part of a ‘post traumatic growth’ (4, 5) during the Corona pandemic. Therefore, we 1) analyzed which changes tumor patients perceived by themselves during the pandemic using the 12-item version of the *Perceptions of Change Scale* (its validation data are presented to underline the instruments´ quality), 2) described how these perceived changes relate to stressors (i.e., perception of burden either due to tumor symptoms or the Corona pandemic restrictions, worries about getting infected) and resources (i.e., meaning in life, spirituality as a resource, awe/gratitude, wellbeing), and 3) identified which of these independent variables would predict these perceived changes using regression analyses. An additional point of interest was how these changes on the one hand and patients´ stressors and resources on the other hand were related to their wellbeing (as a dependent variable).

## Material and Methods

### Recruitment of Patients

Patients with malignant tumors were recruited mainly in eight West and East German centers (Solingen, Wetzlar, Bielefeld, München, Herne, Nordhausen, Jena, Dessau) and a Cancer Self Care group within a five-week time span (from May 6 to June 10, 2020). All patients were assured of confidentiality and were informed about the purpose of the study and data protection information at the starting page of the online survey and at page one of the printed version. Most used the online version, while 50 patients (from Solingen and Jena) used a printed version of the questionnaire. By filling in the anonymous questionnaire, patients consented to participate. Neither concrete identifying personal details nor IP addresses were recorded to guarantee anonymity. The study was approved by the IRB of Jena University Clinic (#5497-04/18; amendment from May 5, 2020). We followed the ethical principles of the Helsinki convention.

As a reference sample for self-perceived changes we enrolled putatively healthy persons within the same time span (anonymous online survey). These were recruited *via* snowball sampling in different networks in Germany, i.e., university students and staff, research collaborators, websites of neighbor dioceses, Facebook sites, etc. from June 9 to June 21. As well, all were invited to spread the information about this survey in their personal networks and websites. Participants were assured of confidentiality and were informed about the purpose of the study and data protection information at the starting page of the online survey. There was no specific incentive, and we had no explicit exclusion criteria.

### Measures

In the following we will describe the perceived changes as dependent variables and influencing stressors and resources as independent variables.

#### Perception of Changes

The COVID-19 pandemic and related social and individual restrictions may have changed people’s specific attitudes, perceptions and behaviors. To assess which changes due to the Corona pandemic were observed, we formulated 13 statements which cover the following topics: more intense relations, perception of nature, times of quietness, loneliness, worrying reflections, and interest in spiritual issues. The respective items were introduced by the phrase “Due to the current situation…”, which referred to the Corona pandemic. Agreement or disagreement was scored on a 5-point scale (0 - does not apply at all; 1 - does not truly apply; 2 - neither yes nor no; 3 - applies quite a bit; 4 - applies very much). The internal consistency of these items will be described in this article. The scores were referred to a 100% level (transformed scale score). Scores > 60% indicate higher agreement (positive attitude/behavior), scores between 40 and 60 indifference, and scores < 40 disagreement (negative attitude/behavior).

A 24-item version of this shortened 12-item version of the *Perceptions of Change Scale* is currently in use (Cronbach´s alpha = .91; 5 factors) in different healthy samples and can be requested for research purposes by the primary author. The short version of this questionnaire is available as **Supplementary Material**.

#### Spiritual and Religious Attitudes in Dealing With Illness (SpREUK-15)

The SpREUK questionnaire was developed to investigate whether or not patients with chronic diseases living in secular societies rely on spirituality as a resource to cope (6, 7). The instrument relies on essential motifs found in counseling interviews with chronic disease patients (i.e., search for a transcendent source to rely on, having trust/faith, reflection of life and subsequent change of life and behavior).

The 15-item SpREUK questionnaire differentiates 3 factors:

1. *Search* (for support/access) deals with patients´ intention to find or have access to a spiritual/religious resource to cope with illness, and having interest in spiritual/religious issues.
2. *Trust* (in higher source) is a measure of intrinsic religiosity dealing with patients´ conviction to be connected with a higher source which carries through, and to be sheltered and guided by this source – whatever may happen.
3. *Reflection* (positive interpretation of situation/disease) deals with cognitive reappraisal and subsequent attempts to change (i.e., reflect on what is essential in life; hint to change life; chance for development; illness has meaning, etc.)

Some phrasings were moderately adjusted in the sense that the phrasing “my illness” (has made me…) was replaced by “the current situation” (has made me…).

The internal consistency of the SpREUK-15 ranges from Cronbach´s alpha = .86 to .91. The items were scored on a 5-point scale from disagreement to agreement (0 - does not apply at all; 1 - does not truly apply; 2 - neither yes nor no; 3 - applies quite a bit; 4 - applies very much). The scores were referred to a 100% level (transformed scale score). Scores > 60% indicate higher agreement (positive attitude), scores between 40 and 60 indifference, and scores < 40 disagreement (negative attitude).

We added a further item (A37) with the same scoring which asks whether faith is a strong hold in difficult times. This item was used as a differentiating variable.

#### Spiritual-Religious Self-Categorization

The SpREUK includes two specific items which ask whether persons regard themselves as a spiritual and/or religious person (without defining what these terms may mean). Scores > 2 indicate agreement and scores < 3 indifference or disagreement. Subsequently one can categorize persons who regard themselves as religious and spiritual (R+S+), religious but not spiritual (R+S-), not religious but spiritual (R-S+) and neither religious nor spiritual (R-S-).

#### Awe and Gratitude (GrAw-7)

To address times of pausing for ‘wonder’ in specific situations (mainly in nature), we measured feelings of wondering awe and subsequent feelings of gratitude as a perceptive aspect of spirituality with the 7-item Gratitude/Awe scale (GrAw-7) (8). This scale has good psychometric properties (Cronbach’s alpha = .82) and uses items such as “I stop and then think of so many things for which I’m really grateful”, “I stop and am captivated by the beauty of nature”, “I pause and stay spellbound at the moment” and “In certain places, I become very quiet and devout”. Thus, awe/gratitude operationalized in this way is a matter of an emotional reaction towards an immediate and ‘captive’ experience. All items were scored on a 4-point scale (0 - never; 1 - seldom; 2 - often; 3 - regularly), referred to a 100-point scale.

#### Meaning in Life (MLQ)

Whether respondents were in search of meaning in life or already had found it, was measured with the 10-item Meaning in Life Questionnaire (MLQ) (9). The 5-item Search subscale uses items such as “I am looking for something that makes my life feel meaningful” and “I am always looking to find my life’s purpose”, and the 5-item Presence subscale items such as “My life has a clear sense of purpose” and “I have discovered a satisfying life purpose.” Internal consistence of both subscales is good to very good (Cronbach´s alpha between .81 and .92). Items are scored form 1 (absolutely untrue) to 7 (absolutely true). The higher the MLQ subscale score, the higher the perceived meaning in life is.

#### Well-Being Index (WHO-5)

To assess participants’ well-being, we used the WHO-Five Well-being Index (WHO-5). This short scale avoids symptom-related or negative phrasings, and measures well-being instead of absence of distress (10). Representative items are “I have felt cheerful and in good spirits” or “My daily life has been filled with things that interest me”. Respondents assess how often they had the respective feelings within the last two weeks, ranging from at no time (0) to all of the times (5). Here we report the sum scores ranging from 0 to 25. Scores < 13 would indicate rather depressive states.

#### Perception of Burden

Perceived daily life affections due to disease related symptoms, feelings of being restricted in daily life by the Corona pandemic, and feelings of being under pressure (i.e., stress and fear) due to the Corona pandemic were measured using three visual analogue scales (VAS), ranging from 0 (not at all) to 100 (very strong).

#### COVID-19 Pandemic Outcomes

Several tumor patients reported that they were “Irritated or unsettled by different statements about the danger and the course of the corona infection in the public media” and that they are “Worrying to be infected with COVID-19 virus and to have complicated course of disease”. Both statements were addressed with two single items. Agreement to these statements was scored from not at all, a little, somewhat and very much.

#### Health Behaviors

Alcohol consumption was scored on a 5-grade scale: never, at least once per month, 2-3 times per month, 1-2 times per week, several times per week. Usage of relaxing drugs, physical activity/sporting, meditation and praying were measured with a 4-grade scale: never, at least once per month, at least once per week, at least once per day.

### Statistical Analyses

Descriptive statistics and analyses of variance (ANOVA) of the influencing and outcome variables (wellbeing, stressors, resources and perceived changes), internal consistency (Cronbach’s coefficient α) and factor analyses (principal component analysis using Varimax rotation with Kaiser’s normalization) of the 12 items of the Perceived Changes Scales as well as first order correlation (Spearman rho) and regression analyses with perceived changes as dependent variables were computed with SPSS 23.0. Given the exploratory character of this study, significance level was set at p <.01. With respect to classifying the strength of the observed correlations, we considered r >.5 as a strong correlation, an r between .3 and .5 as a moderate correlation, an r between .2 and .3 as a weak correlation, and r <.2 as negligible or no correlation.

## Results

### Description of the Sample

We had basic data of 330 people with tumors, among them a fraction responded only to some basic sociodemographic data but not to the wellbeing and burden questions and subsequent other topics. These were regarded as ‘non-responders’ (n=38; 12%). These non-responders did not significantly differ from the responders with respect to gender, age, religious affiliations or tumor stage (data not shown). Nevertheless, among the responders (n=292) not all responded to all questionnaire modules.

As shown in **Table 1**, men (72%) and persons living with a partner (80%) were predominating in the sample. Their mean age was 66.7 ± 10.8 [29-92] years (25% < 60 years, 33% 60-70 years, 41% > 70 years). Patients with prostate cancer (42%) and larynx tumors (17%) were predominating in the sample. Most had a primary tumor (67%) and a progressive state (42%). A large fraction stated they were already treated effectively (47%).

**Table 1:** Sociodemographic data of enrolled patients.

	n	% of responders	mean± SD	range
**Gender**				
Women	81	28		
men	207	72		
**Age (years)**	285		66.7 ± 10.8	29-92
<60 years	72	25		
60-70 years	95	33		
>70 years	118	41		
**Partner status**				
Living with partner	235	80		
Living without partner	57	20		
**Tumor localizations**				
Larynx	55	17		
Breast	34	10		
Prostate	138	42		
Other	60	18		
(no data)	43	13		
**Tumor description**				
Primary tumor	196	67		
Relapse	61	21		
Metastases	66	23		
**Tumor stage**				
Early stages (St. 0-II)	94	32		
Progressive stages (St. III-IV)	124	42		
Unclear stage	74	25		
**Treatments intentions**				
Curative treatment	82	28		
Palliative treatment	42	14		
No active treatment	30	10		
Already treated effectively	138	47		
**COVID-19 tested**				
Positively tested	0	0		
Negatively tested	17	6		
No testing	275	94		
**Irritated or unsettled by different statements about the danger and the course of the corona infection in the public media**			1.7 ± 0.9	0-3
Not at all	30	10		
A little	86	30		
somewhat	102	35		
very much	74	25		
**Worrying to be infected with COVID-19 virus and to have complicated course of disease**			1.7 ± 1.0	0-3
Not at all	36	13		
A little	88	31		
somewhat	92	32		
very much	70	25		
**Religious affiliation**				
Christians	175	60		
Other	14	5		
none	103	35		
**Spiritual-religious self-categorization**				
R+S+	41	16		
R+S-	44	17		
R-S+	16	6		
R-S-	155	61		
n.d.	36	–		
**Faith as strong hold in difficult times**			1.6 ± 1.5	0-4
Disagreement	131	51		
Undecided	41	16		
Agreement	83	33		
**Meditation**				
Never	178	69		
At least once per month	32	12		
At least once per week	31	12		
At least once per day	18	7		
**Praying**				
Never	152	59		
At least once per month	23	9		
At least once per week	34	13		
At least once per day	51	20		
**Wellbeing and burden**				
Wellbeing (WHO5)	286		14.7 ± 6.0	0-25
WHO5 scores < 13	101	35		
WHO5 scores 13-18	88	31		
WHO5 scores > 18	97	34		
Daily life affection due to symptoms (VAS)	289		39.8 ± 26.4	0-100
Restricted in daily life by corona pandemic (VAS)	274		45.1 ± 26.4	0-100
Under pressure due to corona pandemic (VAS)	289		32.1 ± 28.5	0-100
**Meaning in life (MLQ)**				
Search	264		16.1 ± 7.8	0-35
Presence	266		26.5 ± 6.4	0-35
**Spirituality and Coping (SpREUK-15)**				
Search	258		25.5 ± 25.9	0-100
Trust	259		38.8 ± 30.6	0-100
Reflection	260		45.4 ± 25.1	0-100
**Awe/Gratitude (GrAw-7)**	262		57.4 ± 20.2	0-100

A majority had a Christian denomination (60%), a few had other religious orientations (5%), while 35% had no religious affiliation. However, most (61%) regarded themselves as neither religious nor spiritual (R-S-) and 33% as religious (R+S+ or R+S-). 33% agreed that their faith is a strong hold in difficult times, 16% were undecided, and 51% disagreed.

The reference sample (n=993) had a mean age of 52.6 ± 11.2 [31-92] and was thus younger; 33% were men and 67% women. 75% were living in a family household, 21% as singles and 4% in living communities. They were from different professions (17% administration, 14% education, 12% economy, 26% medicine, 31% other).

### Wellbeing, Meaning in Life and Spirituality in the Sample

In the following we describe external measures which are of relevance to describe patients´ wellbeing, meaning in life and spirituality indicators. These analyses are mostly descriptive (mean values and standard deviations), followed by analyses of variance (ANOVA).

Patients´ wellbeing scores were in the lower range (referring to the general range of the WHO5 scale as depicted in **Table 1**), while their perceived daily life affections due to tumor symptoms, and also feelings of being restricted in daily life the by Corona pandemic or feelings of being under pressure due to the Corona pandemic scored in the lower mid-range, however, with large variance (**Table 1**). Wellbeing was significantly higher in older persons (16.2 ± 5.4) as compared to 60-70 years old patients (14.6 ± 5.9) or younger ones (12.1 ± 5.8) (F(2,278)=11.1, p<.0001; ANOVA).

Most (60%) were somewhat to very much irritated or unsettled by different statements about the danger and the course of the COVID-19 infection in the public media, and 57% were somewhat to very much worrying to be infected with COVID-19 virus and to have a complicated course of disease. However, most were so far not tested for a COVID-19 infection (94%), and 6% were negatively tested. None of the respondents was positively tested, only one person in the non-responder group.

Search for meaning in life (MLQ) scored rather low with respect to the scale´s general range, while most already have found meaning in life and thus scored high on MLQ´s Presence component (**Table 1**). Similarly, SpREUK´s Search for a spiritual source scored rather low with respect to the range and interpretation of scores, while SpREUK´s Trust scored higher (in the lower mid-range); SpREUK´s Reflection scale scored in the mid-range. Similarly, the perception of wondering awe in distinct situations and subsequent perceptions of gratitude (GrAW-7 scale) scored in the mid-range (**Table 1**).

### Patients´ Perception of Changes

To better summarize and calculate patients´ perceived changes in attitudes and behavior, an explorative factor analysis of the respective items was performed. A Kaiser-Mayer-Olkin value of .76 (as a measure for the degree of common variance) indicated that the item pool is suited for principal component factor analysis. The item “treating others with more caution” was deleted due to a low factor loading. The 12 remaining items had a good internal consistency (Cronbach´s alpha = .82) and differentiated in four factors that would account for 72% of variance (**Table 2**):

1. *Perception of nature and silence*, with four items and good internal consistency (Cronbach´s alpha = .821): going outdoors more often and perceiving nature more intensely, consciously taking time for silence and enjoying quite times of reflection.
2. *Worrying reflections and loneliness*, with four items and good internal consistency (Cronbach´s alpha = .797): concerned about meaning in life and the lifetime one has, more intense perception of loneliness and feelings of being cut off from life (due to the pandemic restrictions).
3. *Interest in spirituality*, with two items and very good internal consistency (Cronbach´s alpha = .909): praying/meditating more than before, and more interest in religious/spiritual topics as a strategy to cope.
4. *Intense relationships*, with two items and acceptable internal consistency (Cronbach´s alpha = .636): more intensive perceptions of relationships with partner/family and with friends

**Table 2:** Factor and reliability analyses of the 12-item Perceived Changes Questionnaire.

“Due to the current situation I (am) ….”	Mean value	SD	Corrected item – scale correlation	Cronbach´s alpha if item deleted(alpha = .820)	Factor loading			
					1	2	3	4
Eigenvalue					4.3	1.9	1,3	1.1
Cronbach´s alpha					.821	.787	.909	.636
**Factor 1: Perception of nature and silence**								
perceive nature more intensely.	2.64	1.07	.531	.802	.798			
go outdoors much more often.	2.50	1.14	.345	.817	.785			
consciously take more time for silence	2.13	1.13	.610	.795	.750		.300	
enjoy quiet times of reflection.	2.14	1.17	.636	.793	.713		.379	
**Factor 2: Worrying reflections and loneliness**								
more concerned about the lifetime that I have.	2.32	1.25	.477	.806		.822		
more concerned about the meaning and meaning of my life.	2.14	1.25	.573	.798		.774		
feel cut off from life.	1.58	1.24	.385	.815		.764		
perceive times of loneliness more intensely.	1.96	1.15	.551	.800	.454	.586		
treat others with more caution	3.06	0.97	–	–	–	/	–	–
**Factor 3: Interest in spirituality**								
pray/meditate more than before.	1.10	1.29	.479	.806			.921	
more interested in religious/spiritual topics.	1.02	1.20	.490	.805			.898	
**Factor 4: Intense relationships**								
perceive the relationships with my friends more intensely.	2.09	1.09	.263	.823				.852
perceive the relationship with my partner/family more intensely.	2.62	1.06	.334	.817				.804

Principle component analysis (Varimax rotation with Kaiser normalization); rotation is converged in 6 iterations. The four factors explain 71% of variance. Difficulty Index (mean/4) = 0.50; all items are in the acceptable range between 0.2 to 0.8.

/- was deleted due to low factor loading (<0.5).

### Wellbeing, Perceived Burden and Perceptions of Change Within the Sample

We performed analyses of variance to assess the influence of variables such as tumor stage and treatment intentions on patients’ wellbeing, perceived burden and perceptions of change.

Only symptom burden was related to higher tumor stages (Stages III-IV) in trend (37.1 ± 26.2 vs 43.4 ± 26.2; F(1,287)=4.2, p=.042), but not general stress perception due to the COVID-19 pandemic (45.7 ± 24.4 vs 44.6 ± 24.4; F(1,272)=0.1, n.s.) or patients´ wellbeing (14.8 ± 5.8 vs 14.5 ± 6.3; F(1,284)=0.2, n.s.). Patients who were already treated effectively reported higher wellbeing scores than the other patients (13.7 ± 6.0 vs 15.8 ± 5.8; F(1,284)=0.0, p=.003). When patients were treated with a curative intention, their wellbeing was not significantly lower (15.0 ± 5.8 vs 13.7 ± 6.5; F(1,284)=2.5, n.s.), and also palliative treatment intention was not of significant relevance for their wellbeing (14.9 ± 6.0 vs 13.5 ± 5.7; F(1,284)=1.8, n.s.). Instead, palliatively treated patients were in trend more affected by their symptoms (38.2 ± 26.4 vs 49.5 ± 24.6; F(1,297)=6.6. p=.011) and stronger by the restrictions during the lockdown (43.5 ± 25.9 vs 54.6 ± 28.1; F(1,272)=6.0. p=.015).

To analyze differences in perceived changes as dependent variables which are related to sociodemographic (gender, age, partner status) and tumor related variables (tumor stage and treatment intentions) as independent variables, we performed analyses of variance. Here, the most frequently perceived changes were *Perception of nature and silence* and also *Intense relationships* (**Table 3**). Here, experience of nature and more intensive relations with partner/family were most relevant (**Table 2**). Nevertheless, *Worrying reflections and loneliness* were also perceived (particularly being more concerned about the lifetime one has), while *Interest in spirituality* scored lowest. There were no significant differences related to gender and age groups, but a weak impact of living with or without a partner on the perception of *Intense relationships* (**Table 3**). Compared to a reference sample of putatively healthy (non-tumor) persons recruited in the same time span, *Perception of nature and silence* scored identically, while *Worrying reflections and loneliness* and *Intense relationships* were in a similar range; in contrast, *Interest in spirituality* scored much lower in tumor patients (**Table 3**). We performed no statistical analyses regarding whether these differences were significantly different or not, as this was not the objective of this study.

**Table 3:** Expression of change perceptions within the sample of tumor patients and a reference sample of healthy persons.

		Perception of nature and silence	Worrying reflections and loneliness	Interest in spirituality	Intense relationships
Reference sample*	mean	58.28	45.78	39.56	62.18
	SD	24.39	23.12	27.68	21.62
**Tumor patients**	mean	58.88	50.13	26.57	58.98
	SD	22.89	23.85	29.59	23.08
**Partner status**					
Living without partner	mean	59.38	50.71	27.90	53.35
	SD	21.42	23.61	30.15	25.62
Living with partner	mean	58.76	49.99	26.25	60.34
	SD	23.28	23.96	29.51	22.27
F(1,285-288) values		0.03	0.04	0.14	4.19
p values		n.s.	n.s.	n.s.	.042
**Wellbeing (WHO-5)**					
Scores < 13	mean	60.28	62.79	31.75	59.22
	SD	23.53	19.53	31.40	22.20
Scores 13-18	mean	59.82	49.57	22.38	61.21
	SD	20.78	22.68	28.19	22.23
Scores > 18	mean	56.23	37.78	24.87	56.44
	SD	23.98	22.85	28.30	24.48
F(2,282-284) values		1.08	32.80	2.58	1.00
p values		n.s.	<.0001	.077	n.s.
**SpR self-categorization**					
R-S-	mean	54.78	46.40	11.77	57.98
	SD	22.24	23.50	19.07	22.96
R+S+/R+S-/R-S+	mean	61.59	52.06	47.75	58.66
	SD	22.88	22.94	29.21	23.43
F(1,253-254) values		5.60	3.62	141.66	0.05
p values		.019	.058	<.0001	n.s.
**Faith as a strong hold**					
No	mean	52.83	44.02	9.83	58.02
	SD	22.82	23.89	18.35	24.17
Indifferent	mean	62.65	56.30	28.66	60.67
	SD	18.93	21.62	27.70	20.83
yes	mean	64.33	53.16	50.46	58.99
	SD	22.81	23.15	28.40	22.76
F(2,251-252) values		7.79	6.33	74.68	.021
p values		.001	.002	<.0001	n.s

*Healthy persons recruited in a similar time span (N=993; mean age of 52.6 ± 11.2 [31-92]; 33% men and 67% women).

In the sample of tumor patients there were no significant differences in these perceptions with respect to their tumor stage (data not shown). However, those who were not treated actively anymore had significantly higher *Worrying reflections and isolation* scores than the others (61.5 ± 18.1 vs 48.8 ± 24.1; F(1,288)=7.7, p=.006).

Patients´ wellbeing was significantly related to the perception of *Worrying reflections and loneliness* which was highest in the group of patients with WHO5 scores < 13, indicating depressive states (**Table 3**).

The spiritual/religious self-categorization had a significant impact on *Interest in spirituality* and *Perception of nature and silence* which scored lowest in R-S- persons. Those who had access to faith as a resource in difficult times had significantly higher *Interest in spirituality*, *Perception of nature and silence*, and *Worrying reflections and loneliness* scores (**Table 3**).

### Associations Between Perceptions of Change With Indicators of Wellbeing, Meaning in Life, and Spirituality

Next we performed correlation analyses to assess how the putative stressors and resources (i.e. wellbeing, meaning in life and spirituality) as dependent variables related to the perceptions of changes. The respective ordinal scales are not normally distributed (as tested with the Shapiro-Wilk test) and thus we used the Spearman rho test.

The four perceptions of change factors were moderately interconnected, particularly *Worrying reflections and loneliness* was positively related to *Interest in spirituality* and *Perception of nature and silence* (**Table 4**).

**Table 4:** Correlations between perceived changes and indicators of spirituality, meaning in life, wellbeing and health behaviors.

	Perception of nature and silence	Worrying reflections and loneliness	Interest in spirituality	Intense relationships
**Spiritual transformation**				
Perception of nature and silence	1.000			
Worrying reflections and loneliness	.377^**^	1.000		
Interest in spirituality	.323^**^	.413^**^	1.000	
Intense relationships	.358^**^	.221^**^	.086	1.000
**Spirituality**				
Search (SpEUK-15)	.179^**^	.257^**^	.731^**^	.023
Trust (SpEUK-15)	.240^**^	.180^**^	.678^**^	.003
Reflection (SpEUK-15)	.409^**^	.315^**^	.441^**^	.224^**^
Faith as hold in difficult times (A37)	.284^**^	.222^**^	.668^**^	.008
Awe/Gratitude (GrAw-7)	.407^**^	.162^**^	.385^**^	.135
Meditation frequency	.096	.114	.322**	.023
Praying frequency	.248**	.146	.630**	-.026
**Meaning in life**				
Meaning in life - Search	.155	.447^**^	.286^**^	.061
Meaning in life - Presence	.053	-.229^**^	-.051	.157
**Wellbeing**				
Wellbeing (WHO-5)	-.052	-.450^**^	-.075	-.032
Daily life affections through tumor symptoms (VAS)	.095	.331^**^	.088	.059
Daily life restrictions because of Corona pandemic (VAS)	.067	.419^**^	.159^**^	.142
Under pressure (i.e. stress/anxiety) because of Corona pandemic (VAS)	.194^**^	.510^**^	.172^**^	.196^**^
Irritated or unsettled by different statements about the danger and the course of the corona infection in the public media?	.152^**^	.322^**^	.044	.098
Worrying to be infected with COVID-19 virus and to have complicated course of disease	.131	.334^**^	.080	.044
**Current health behaviors**				
Relaxing drugs	.088	.164**	.061	-.058
Alcohol consumption	-.164**	-.036	.071	.014
Physical activity/sporting	.088	-.057	-.006	.039

**p<.0001 (Spearman rho); moderate to strong correlations were highlighted (bold).

*Perception of nature and silence* was moderately related to SpREUK´s Reflection and also to awe/gratitude, and weakly with faith as hold, SpREUK´s Trust, and with the frequency of praying (**Table 4**), indicating that both the perceptive and the cognitive aspects of spirituality were related to this experiential factor.

*Worrying reflections and loneliness* was strongly associated with feelings of being under pressure (i.e. stress/anxiety) because of the Corona pandemic, moderately positively with other indicators of burden and low wellbeing (**Table 4**), and further with SpREUK´s Reflection, MLQ´s Search for meaning in life, and with irritations by different statements about the danger and the course of the COVID-19 infection in the public media, and also with patients´ worries about their own infection with the virus and to have a complicated course of the disease.

*Interest in spirituality* was strongly related with SpREUK´s Search and Trust scales and with faith as hold, moderately with other indicators of spirituality, and weakly also with MLQ´s Search for meaning in life (**Table 4**).

*Intense relationships* was weakly related only to SpREUK´s Reflection scale and with feelings of being under pressure (i.e. stress/anxiety) because of Corona pandemic, but with none of the other variables (**Table 4**).

With respect to health behaviors, physical activity/sporting was not relevantly related to the four change factors (**Table 4**). Alcohol consumption was marginally negatively related to *Perception of nature and silence*, while usage of relaxing drugs was marginally positively associated with *Worrying reflections and loneliness*.

### Predictors of Patients´ Perceived Changes

There are several variables which were significantly associated with the changes tumor patients did perceive during the Corona pandemic. To analyze which of these independent variables could be regarded as predictors of perceived changes (as dependent variables), we performed stepwise regression analyses with significantly related variables. The best fitting model for each of the four dependent variables is depicted in **Table 5**.

**Table 5:** Stressors and resources as independent predictors of perceived changes as dependent variables (stepwise regression analyses).

	Beta	T	p
**Dependent variable: Perception of nature and silence** Model 4: F=20.6, p<0.0001; R^2^=.27			
(constant)		3.221	.002
Awe/Gratitude (GrAw-7)	.331	5.004	<.0001
Meaning in Life - Search (MLQ)	.155	2.631	.009
Reflection (SpREUK)	.180	2.669	.008
Worrying to be infected with COVID-19 virus and to have complicated course of disease	.123	2.107	.036
**Dependent variable**: **Worrying reflections and loneliness** Model 5: F=43.8, p<0.0001; R^2^=.50			
(constant)		4.180	<.0001
Under pressure (i.e. stress/anxiety) because of Corona pandemic (VAS)	.205	2.855	.005
Meaning in Life - Search (MLQ)	.333	6.542	<.0001
Reflection (SpREUK)	.208	2.268	<.0001
Wellbeing (WHO-5)	-.202	-3.388	.001
Daily life restrictions because of Corona pandemic (VAS)	.139	2.104	.036
**Dependent variable: Interest in spirituality** Model 5: F=84.4, p<0.0001; R^2^=.66			
(constant)		-3.099	.002
Search (SpREUK)	.498	8.774	<.0001
Praying	.238	4.369	<.0001
Meaning in Life - Search (MLQ)	.128	3.130	.002
Daily life restrictions because of Corona pandemic (VAS)	.103	2.549	.011
SpR self-categorization	.133	2.206	.028
**Dependent variable: Intense relationships** Model 4: F=9.6, p<0.0001; R^2^=.15			
(constant)		3.917	<.0001
Reflection (SpREUK)	.311	4.203	<.0001
Trust (SpREUK)	-.174	-2.389	.018
Meaning in Life - Presence (MLQ)	.172	2.718	.007
Under pressure (i.e. stress/anxiety) because of Corona pandemic (VAS)	.184	2.927	.004

As shown in **Table 5**, *Perception of nature and silence* was predicted best by awe/gratitude and further by patients’ search for meaning in life, with their ability to reflect their life concerns, and with worry about being infected. These four predictors would explain 27% of variance.

*Worrying reflections and loneliness* was predicted best by patients’ search for meaning in life and by feelings of being under pressure because of the Corona pandemic, and further by their ability to reflect, by low wellbeing, and perceived daily life restrictions because of Corona pandemic. These five predictors explain 50% of variance.

*Interest in spirituality* was predicted best by patients’ search for an access to a spiritual source and by frequency of praying, and further by search for meaning in life, perceived daily life restrictions because of the Corona pandemic, and by a spiritual/religious self-categorization. These five predictors explain 66% of variance.

*Intense relationships* were explained with weak predictive power (R^2^=.15) by patients’ ability to reflect life concerns, low religious Trust, by presence of meaning in life, and by feelings of being under pressure because of the Corona pandemic. However, living with or without a partner had no significant influence.

### Predictors of Patients’ Wellbeing

Are these perceived changes contributing to patients’ wellbeing? Regression analyses revealed that *Worrying reflections and loneliness* (Beta = -.51, T = -8.8, p<.0001) and in trend also *Perception of nature and silence* (Beta = .15, T = 2.5, p=.012) would predict wellbeing (as depending variable), albeit with weak predictive power (R^2^=.22) The first variable would explain 20% of variance and the second would add 1.8% only and is thus irrelevant.

Adding meaning in life, spirituality as a resource, fears and worries, and age as independent variables to the model resulted in six predictors of wellbeing as dependent variable (R^2^=.57), daily life affections due to symptoms (Beta = -.35, T = -7.1, p<.0001; explains 34% of variance), being under pressure due to the Corona pandemic (Beta = -.26, T = -4.7, p<.0001; +12% of explained variance), MLQ´s Presence component (Beta = .17, T = 3.8, p <.0001; +4% of explained variance), religious Trust (Beta = .14, T = 3.2, p<.0001 =.002; +2% of explained variance), *Worrying reflections and loneliness* (Beta = -.22, T = -4.0, p<.0001; +2% of explained variance), and age (Beta = .16, T = 3.5, p=.001; +2% of explained variance). Here, praying, SpREUK´s Search and Reflection scales, and awe/gratitude had no significant influence in this model.

## Discussion

This survey among tumor patients who have to cope with the restrictions of the COVID-19 pandemic revealed that a majority was irritated by different statements about the danger and the course of the Corona infection in the public media, and feared their own infection with the COVID-19 virus. Their wellbeing was rather low and their burden in a mid-range, indicating that they felt moderately restricted in their daily life and under pressure by stress and fear. In fact, 35% had WHO5 scores < 13, indicating depressive states. Patients’ wellbeing was significantly higher in older persons and low in younger ones. Wellbeing was predicted best by a mix of disease and pandemic related variables, and available resources. Their perceived daily life affections due to symptoms alone explained 34% of variance, feelings of being under pressure due to Corona pandemic added further 12%, having found meaning in life added further 4%, while religious Trust 2% and also, *Worrying reflections and loneliness* and also higher age would add together further 6% of explained variance.

Because of the restrictions, patients noticed changes in their attitudes and behaviors. These refer mainly to more intense relationships with partners, family and friends on the one hand, and a more intense perception of nature with more frequent time outside (related to time for silence and enjoying quiet times of reflection) on the other hand. Nevertheless, worrying thoughts (particularly being concerned about the lifetime one has) and perceptions of loneliness were of relevance, too. In contrast, more interest in spiritual issues was of relevance only for some patients. Faith as a hold in difficult times was stated by 33% of patients analyzed herein; most would regard themselves as R-S- and thus it is comprehensible that this resource is of less relevance to most of them.

The observed perceptions of change were similar in women and men and in the different age groups, and not different with respect to patients’ tumor stage. Nevertheless, it is worth mentioning that the few patients (10%) who were not treated actively anymore had significantly higher *Worrying reflections and isolation* scores than the other ones; these are still in contact with their oncologists, but obviously in fear. Compared to a reference sample of healthy persons recruited in the same time span, *Perception of nature and silence* scored identically, while *Worrying reflections and loneliness* were slightly higher and *Intense relationships* were slightly lower in tumor patients compared to the healthy reference sample, but in a similar range; in contrast, *Interest in spirituality* was much lower in tumor patients. Thus, tumor patients (and also healthy persons) perceived similar changes of their attitudes and behaviors, with the exception of *Interest in spirituality*. A reason for this lower interest in spiritual issues in enrolled tumor patients could be the predominance of women in the healthy sample who are generally more spiritually interested than men.

The relevant predictors of the perceived changes of attitudes and behaviors were complex. Pausing to wonder and stand still in silence in specific situations (awe) as an aspect of perceptive spirituality was the best predictor of *Perception of nature and silence*. This means patients became more aware of their surroundings, particularly that they used the time of restriction to go into nature and perceive it more consciously. Related as a predictor was the ability to reflect life concerns, to reflect on what is essential in life, and to change aspects of life. In the same vain was the finding that patients’ search for meaning in life was a further predictor. This time-out phase thus encouraged reflection processes and more awareness (‘mindfulness’).

Search for meaning in life was the best predictor of patients´ *Worrying reflections and loneliness*, which was further predicted by the feeling of being under pressure because of the Corona pandemic. The COVID-19 restrictions obviously left some patients in the situation that they had difficulties in adequately coping and in finding meaning. In fact, the ability to reflect on one’s own life concerns was a further predictor, indicating an inner process of clarification and prioritization to cope with these worries and feelings of isolation. Other, yet weaker predictors were low wellbeing, and perceived restrictions of life due to the pandemic.

Although *Interest in spirituality* was relevant only for a fraction of persons, it is nevertheless a relevant resource to cope also in secular societies (6, 11–13). These perceived changes were predicted best by patients´ search for access to a spiritual source and by their frequency of praying. In line with this, searching for meaning in life was an additional (yet weaker) predictor. More relevant as a further predictor was praying (20% of patients were praying at least once per day). Praying means to be in ‘communication’ with God as an external source of help, to let go fears and worries, to ask for help and to express trust when other resources seem to be less helpful (14–16).

*Intense relationships* were explained with low predictive power by patients´ ability to reflect their life concerns, and further by low religious Trust (which would underline the aforementioned statement that referring to God might be an ‘alternative’ when stable partner relations are experienced as less helpful), having found some meaning in life, and feelings to be under pressure (i.e. stress/anxiety) because of Corona pandemic. However, these predictors explain only 15% of variance, and thus we do not consider them to be of central importance; therefore, other unidentified variables might be of relevance.

Gender or age were not of relevance for any of these changes in perceptions. Further, patients´ health behaviors were of marginal relevance only. Of interest was that the usage of relaxing drugs was at least marginally positively associated with *Worrying reflections and loneliness*. This would indicate that for some patients the COVID-19 restrictions were more severe than for others and they required medication. Further, alcohol consumption was marginally negatively related to *Perception of nature and silence*, indicating that the ability to go out and perceive nature and experience times of quietness may prevent alcohol consumption. However, this alcohol consumption was marginally negatively related to more intensive perception of loneliness, and thus it is not a relevant indicator of loneliness.

It is obvious that several tumor patients have changed their attitudes and behavior. These can be seen as indicators of ‘posttraumatic growth’ (4, 5) due to the Corona lockdown experience. However, are these perceived changes also contributing to their wellbeing? It was striking that 35% of tumor patients had wellbeing scores < 13, 31% had moderate and 34% high wellbeing. In the healthy reference sample recruited in a similar period, we found 28% with scores <13, 39% with moderate wellbeing and 33% with high wellbeing. Thus, also healthy persons are emotionally affected by the Corona pandemic restrictions. The wellbeing groups differ only with respect to tumor patients´ *Worrying reflections and loneliness*. Nevertheless, post-hoc analyses showed that the ‘depressive states’ patients felt significantly (p<.0001) more affected in their daily life situation by their symptoms (mean 55.1 ± 24.7; F(2,282)=43.4), by the Corona restrictions (mean 59.1 ± 23.7; F(2,267)=36.0), and felt under pressure because of the Corona pandemic (mean 50.3 ± 29.4; F(2,282)=52.1) compared to the other wellbeing groups. Regression analyses revealed that *Worrying reflections and loneliness* and in trend also *Perception of nature and silence* would predict wellbeing to some extent (R^2^=.22). Adding meaning in life, spirituality as a resource, fears and worries and age changed the prediction model in as much as now patients´ wellbeing was predicted with stronger power (R^2^ =.57) by a mix of disease and pandemic related stressors, and available resources (meaning in life and religious trust).

What are the consequences from these findings for the psycho-oncological support of patients, both in the COVID-19 pandemic (which is not yet ‘solved’) and also for future difficult situations because of restrictions? – When perception of nature and peaceful silence and wondering awe are a resource for several tumor patients, one has to consider specific offers to experience these, either in a group (to avoid feelings of isolation and loneliness) or individually. These could be guided forest walks (17, 18), also with the option of virtual walks (which could be considered for specific groups at risk) to encourage feelings of inner peace and stress-relief. A further option would be mindful mediation (19, 20), as both individual offers at home and also in group settings; even web-based mindfulness approaches seem to be effective (21). For several patients, their faith was a resource to cope, and thus retreating in monastic contexts to sensitize for the topic of spirituality or consolidate faith might be an option. This would also allow talks with pastoral professionals when phases of religious struggles (22, 23) or spiritual dryness (24, 25) may affect patients´ emotional and spiritual wellbeing. Here, patients´ spiritual needs should be assessed to support them in the requirements they express (26–29). Gonçalves et al. (30) suggested that during the Corona pandemic the “use of spirituality” could be a tool to promote mental health particularly in psychiatric patients. However, in our study with tumor patients most had no specific interest in spiritual or religious issues, but were nevertheless perceiving awe in specific situations. These perceptions could be sensitized by awareness training. In this sample, the experience of awe and gratitude scored significantly higher in women compared to men (F=9.7, p=.002), and thus they might be especially suited.

During the COVID-19 pandemic several patients required intensive care treatment and were isolated from their relatives. Reports from oncologists as well as ICU staff and patients´ relatives underlined that the restrictions (with either no or minimal contact only) were causing mental and spiritual pain on the side of the patients, their relatives, but also on the staffs´ side (1, 31). When it is true that mentally stabilizing and supportive relations with partners, family and friends are that important, one has to consider possibilities to facilitate contacts with the family. Here, digital media facilities to connect isolated patients with their relatives were often used, particularly in such departments. Furthermore, it is necessary to develop ways to remain in personal contact within families during crisis times.

Physicians and psychologists are mostly able to treat depression. However, during the Corona crisis we have to prevent and/or overcome demoralizations of patients, physicians, and their staff (32). Here, an additional planned integration of structured access to spiritual care seems to be important, not only for the field of cancer care.

### Limitations

This study was planned as an online survey and thus persons without internet access may not be reached adequately. Nevertheless, some have used to option to fill a concrete (paper-pencil) questionnaire. The sample might not be representative for all tumor patients in Germany, as we recruited in distinct centers related to members of the AG PRIO within the German Cancer Society. However, we enrolled centers from East and West Germany to balance putative differences in socialization and cultural peculiarities.

The untypical predominance of male persons (72%) in such a survey, with specific tumor localizations (i.e., prostate and larynx), can be attributed to our recruiting centers with their specific specialization. Studies enrolling more women with their specific tumor localizations are needed.

Further, we have no information about the reasons of those who have not participated. At least we were able to compare persons who have provided basic socio-demographic data but decided not to finalize the online questionnaire with those who completed the survey. Here, no significant differences with respect to gender, age, religious affiliations or tumor stage were found.

The most important limitation might be that patients´ perceived changes of their attitude and behaviors were assessed ‘retrospectively’ by themselves. For them, these perceptions are important and for researchers informative to provide additional support. However, longitudinal studies are required to substantiate patients´ perceptions.

### Outlook

The majority of patients with malignant tumors are not necessarily hospitalized and not all have access to psychological or pastoral support which may help them to cope with their fears and worries, particularly during the Corona pandemic with its individual and social restrictions. To overcome feelings of isolation, depressive states, and insecurity about future perspectives, further supporting offers are needed, particularly in their socio-spatial surrounding where patients are mostly left alone. In this study among tumor patients from a secular society the topics of meaning in life, having trust, stable relationships, mindful encounter with nature, and times of reflection were important topics. These are the domains of psychotherapy and spiritual care. Particularly in secular societies, non-religious forms of (secular) spirituality are relevant (29). Spirituality, understood in this more broad and open context (33), can be seen as an individual resource for patient’s resilience, which is “maintaining self-esteem, providing a sense of meaning and purpose, giving emotional comfort and providing a sense of hope” (34) in personal crisis management. Such spiritual care approaches (27, 35) can be easily incorporated into a more comprehensive treatment and support of tumor patients, particularly in times of pandemic restrictions.

## Data Availability Statement

The raw data supporting the conclusions of this article will be made available by the authors, without undue reservation.

## Ethics Statement

The studies involving HUMAN PARTICIPANTS were reviewed and approved by the Ethic Committee of the Jena University Clinic, Bachstraße 18, 07743 Jena, Germany (#5497-04/18; amendment from May 5, 2020). Written informed consent for participation was not required for this study in accordance with the national legislation and the institutional requirements.

## Author Contributions

The study was initiated by JB. JH, SW, WG, and JB organized the distribution of the survey. Data analysis was performed by AB. The *Perceptions of Change* scale was developed by AB. The first draft of the manuscript was written by AB and JB. All authors contributed to the article and approved the submitted version.

## Conflict of Interest

The authors declare that the research was conducted in the absence of any commercial or financial relationships that could be construed as a potential conflict of interest.
