# Supplementary material for: Tumor Patients´ Perceived Changes of Specific Attitudes, Perceptions, and Behaviors Due to the COVID-19 Pandemic and Its Relation to Reduced Wellbeing
Source: Front Psychiatry. 2020 Oct 9;11:574314. doi: 10.3389/fpsyt.2020.574314 (PMC7581913; doi:10.3389/fpsyt.2020.574314)
Supplement: Supplementary file 1 [file DataSheet_1.pdf]

	The consequences of the corona pandemic are changing many of our behaviors, attitudes, and feelings. That may or may not be the case with you. Please check to what extent the following statements are currently applicable to you.	does not apply at all	does not really apply	neither yes nor no	applies quite well	definitely applies
	Due to the current situation ...					
1	I perceive the relationship with my partner / family more intensely.	0	1	2	3	4
2	I perceive the relationships with my friends more intensely.	0	1	2	3	4
10	I go outdoors much more often.	0	1	2	3	4
11	I perceive nature more intensely.	0	1	2	3	4
12	I consciously take more time for silence.	0	1	2	3	4
13	I enjoy quiet times of reflection.	0	1	2	3	4
14	I perceive times of loneliness more intensely.	0	1	2	3	4
15	I feel cut off from life.	0	1	2	3	4
23	I am more concerned about the lifetime that I have.	0	1	2	3	4
24	I am more concerned about the meaning and purpose of my life.	0	1	2	3	4
29	I pray / meditate more than before.	0	1	2	3	4
20	I am more interested in spiritual / religious issues.	0	1	2	3	4

12-item short version of the *Perceptions of Change Scale* © Prof. Dr. Arndt Büssing, Witten/Herdecke University (email: Arndt.Buessing@uni-wh.de)
